# Temporal trend in hospitalizations for malignant neoplasm and benign neoplasm: a nationwide study, China, 2004–2020

**DOI:** 10.1186/s12885-024-11866-x

**Published:** 2024-01-30

**Authors:** Xinqiang Zhang, Yuanyuan Li, Guifang Zhang, Changsheng Ma, Bo Liu, Yong Yin

**Affiliations:** 1 Department of Radiophysical Technology, Shandong Cancer Hospital, No.440, Jiyan Road, Jinan City, Shandong province 250000 China; 2 Statistical Analysis Department, Clinical Research Center, Qilu Pharmaceutical Co., Ltd, 8888 Lvyou Street, Jilin, 250102 China

## Abstract

The increasing cancer burden calls for reliably assessed changes in the hospitalizations for tumors over time and space in China. This study evaluated trends in hospitalization rate, in-hospital mortality, length of stay (LOS), and medical costs for malignant and benign neoplasms. Data were derived from China Health Statistical Yearbooks from 2004 to 2020. Temporal trends in hospitalization rates and in-hospital mortality rates were assessed through the Cochran-Armitage Test. We used the linear model with continuous variables to test for the trend. The malignant neoplasm hospitalization rate increased from 1.1‰ to 5.8‰ and the benign neoplasm increased from 1.0‰ to 2.0‰. The in-hospital mortality rate due to malignant neoplasm and benign neoplasm decreased from 5.11 to 2.87% (P for trend < 0.001) and 0.14–0.01% (P for trend < 0.001), respectively. Among all patients hospitalized with malignant neoplasm, the average cost per hospitalization significantly increased during the study period (P for trend < 0.001), adjusted for the Consumer Price Index. However, the average LOS gradually decreased (P for trend < 0.001). In line with the trend of malignant neoplasm, the average cost per hospitalization increased significantly among all patients hospitalized for benign neoplasm (P for trend < 0.001), and the average LOS showed a steady downward trend (P for trend < 0.001). We found upward trends in hospitalization rates, and medical costs in neoplasms. By contrast, substantial decreases in in-hospital mortality and LOS. The hospitalization rate gap between urban and rural areas is narrowed.

## Introduction

Cancer is one of the major public health problems and is a leading cause of death, placing an increasing economic burden on cancer in China over the past half-century [1–3]. In 2020, China alone accounted for a quarter of the world’s cancer deaths [4]. Due to rapid economic and social development, the major cancer pattern in China is changing from a developing country to a developed country. There was a huge variation in cancer incidence and mortality in China, the United States, and the United Kingdom in 2020. The incidence of cancers in China (204.80 per 100,000 population) was lower than those in the United States (362.20 per 100,000 population) and the United Kingdom (319.90 per 100,000 population). However, China (129.40 per 100,000 population) had much higher mortality than the United States (86.30 per 100,000 population) and the United Kingdom (100.50 per 100,000 population) [5]. In China, during the planned economy era from 1949 to 1978 and the early period of reform and opening up from 1979 to 1993, urban people were mainly engaged in industrial and commercial work, while rural people mostly lived on farms. Since China entered the era of a market economy in 1993, the gap between urban and rural areas in socioeconomic status has gradually narrowed [6]. Hospitalization is related to the decline in quality of life and the increase in costs of care. The length of stay (LOS) and hospital cost are the key indicators to reflect the burden of hospitalization and the demand for beds, medical staff, and the comprehensive medical service capacity of the hospital to a certain extent.

Several studies have examined the time trend in incidence rate, mortality rate, and financial burden for neoplasm at regional and national levels in China [1, 7–8]. However, few studies have assessed changes in the hospitalizations for neoplasms over time and space in China. This study evaluated trends in hospitalization rate, in-hospital mortality, LOS, and hospital charge on malignant neoplasm and benign neoplasm from 2004 to 2020. The findings of this study help understand the malignant neoplasm and benign neoplasm hospitalizations and associated hospital outcomes, thereby providing evidence for decision-makers to prioritize resources and implement preventive measures against neoplasm.

## Materials and methods

Data for this study from January 1, 2004, to December 31, 2020, were derived from China Health Statistical Yearbooks, which contain data on health development and population health levels of 31 provinces, autonomous regions, and municipalities directly under the central government, as well as national statistical data for historically important years. The data on medical services in this yearbook used in this study come from the National Statistical Survey on Health Resources and Medical Services, which is a complete survey. The survey includes basic information on medical and health institutions, operation of medical institutions, basic information on health manpower, plans for the demand of health personnel, allocation of medical equipment, discharged patients, demographic information on the whole population, and implementation of health reform measures [9]. From 1999 to 2007, the information on health resources and medical services was provided in the form of paper-based statistical questionnaires submitted by medical and health institutions (except clinics and village health offices) or local health administration departments [10]. Since 2007, the information has been submitted electronically through the provincial platform of the “National Health Statistics Network Direct Reporting System” [10]. The raw data are approved annually for publication by the National Health Commission of China. Disease outcomes for hospitalized patients from 2003 to the present were aggregated by hospitals affiliated with health departments at all levels using the International Classification of Diseases, Tenth Revision (ICD-10). However, before 2003, ICD-9 was used. In addition, medical costs per capita for neoplasm hospitalizations were not published in the 2003 China Health Statistics Yearbook. Considering data availability and continuity, we chose the data from 2004 to 2020. We analyzed trends in the hospitalization rate, in-hospital mortality rate, LOS, and medical cost. Hospitalization rate refers to the ratio of the number of patients hospitalized for neoplasm in a year to the population surveyed in that year. We performed subgroup analyses by age groups and areas. We divided the patients into five age groups: ≤4 years old, 5–14 years old, 15–44 years old, 45–59 years old, and ≥ 60 years old. The location of hospitalization was identified by areas (urban, rural). Urban and rural areas are distinguished according to administrative districts, urban areas include countries and prefecture-lever areas, and rural areas include counties and county-level areas. Continuous variables were expressed as mean ± standard deviation, while categorical variables were expressed as proportions. Temporal trends in hospitalization rates and in-hospital mortality rates across levels of an ordinal variable (e.g., calendar years) were assessed through the Cochran-Armitage Test [11, 12]. We used the linear model with continuous variables to test for the trend [13]. To facilitate direct comparisons of medical costs per capita of patients hospitalized for neoplasm across the years, we converted all costs to 2020 China Yuan using the Consumer Price Index (CPI) [9]. Because the purpose of the study was to examine the temporal trends rather than the causal relationship between calendar years and outcomes, we did not adjust for covariates. All probability values were from 2-tailed tests, and *P* < 0.05 was deemed statistically significant. All statistical analyses were performed with R software (version 4.2.2; R Foundation for Statistical Computing, Vienna, Austria).

## Results

### Demographic characteristics

In China, malignant neoplasm resulted in 31,792,217 hospitalizations and 1,002,561 deaths between 2004 and 2020, and benign neoplasm caused 14,831,103 hospitalizations and 6717 deaths. There were more inpatients in urban areas than in rural areas. The largest proportion of hospitalizations was for patients with malignant neoplasm aged 60 or older (53.23%) and patients with benign neoplasm aged 15 to 44 (43.91%). The mean of medical costs per malignant neoplasm- and benign neoplasm-related hospitalization was ￥14878.92 ± 3944.565 and ￥8957.1 ± 2442.105 respectively. The mean LOS was 14.09 ± 1.426 days for malignant neoplasm and 8.70 ± 1.113 days for benign neoplasm, and urban is longer than rural (Table 1).

**Table 1:** Characteristics of patients admitted for malignant neoplasm and benign neoplasm

discharge		Malignant Neoplasm	Benign Neoplasm
N	all	31,792,217	14,831,103
	Urban, n(%)	22,917,521(72.09%)	10,526,751(70.98%)
	Rural, n(%)	8,874,696(27.91%)	4,304,352(29.02%)
Age distribution (years old)			
	≤ 4	0.55%	1.72%
	5–14	0.69%	1.90%
	15–44	13.26%	43.91%
	45–59	32.32%	38.02%
	≥ 60	53.23%	14.45%
Duration of stay in days, mean (SD)			
	all	14.09(1.426)	8.70(1.113)
	Urban	14.40(1.491)	8.89(1.170)
	Rural	12.57(0.951)	7.98(0.729)
Deaths in hospital, n(%)		1,002,561(3.15%)	6717(0.05%)
Average medical costs per capita (￥)		14878.92(3944.565)	8957.1(2442.105)

### Trends in malignant neoplasm and benign neoplasm-related hospitalizations and in-hospital mortality

**Fig. 1:**
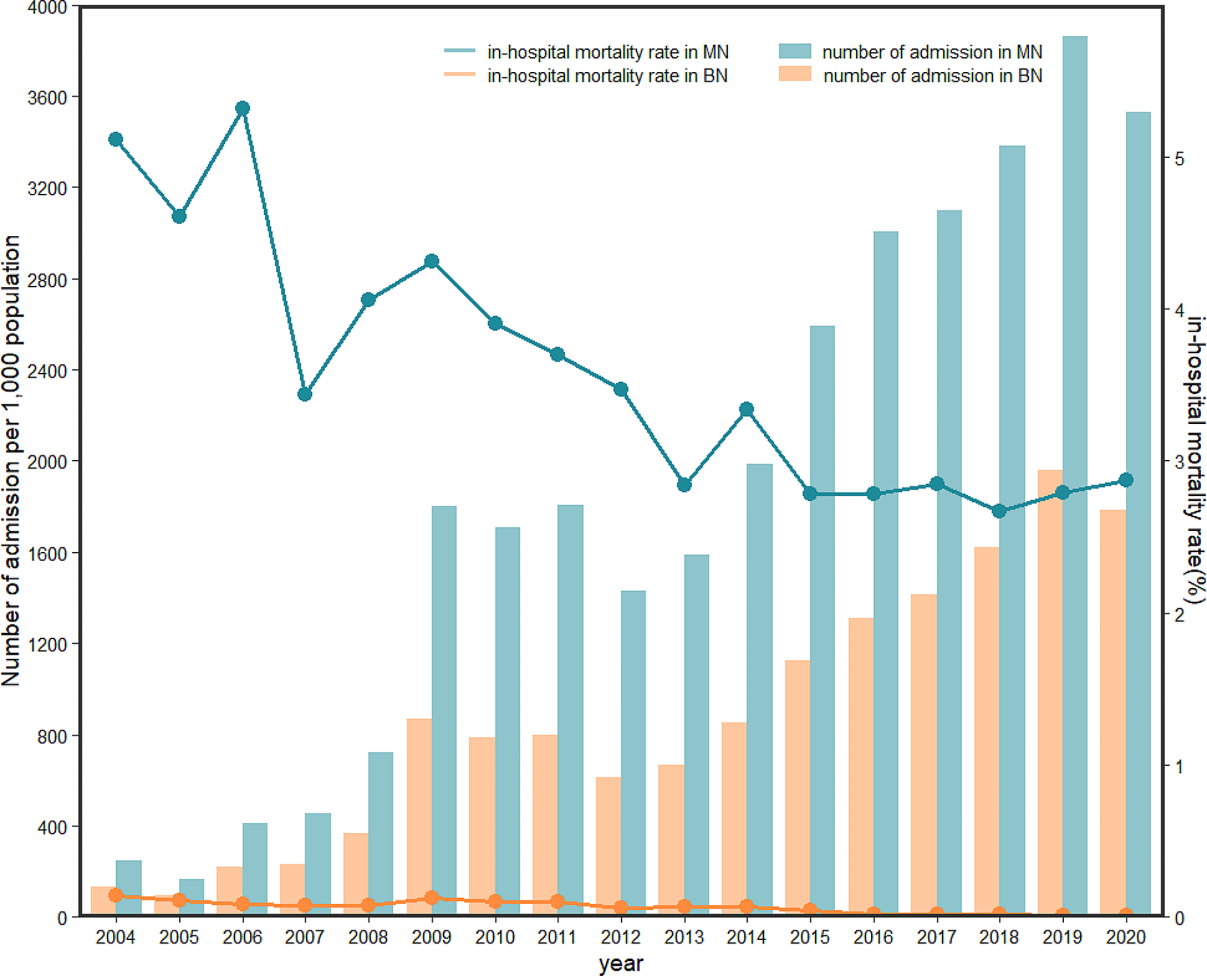
Trends of the number of hospitalizations and in-hospital mortality rate in malignant neoplasm and benign neoplasm-related. MN, malignant neoplasm; BN, benign neoplasm

**Fig. 2:**
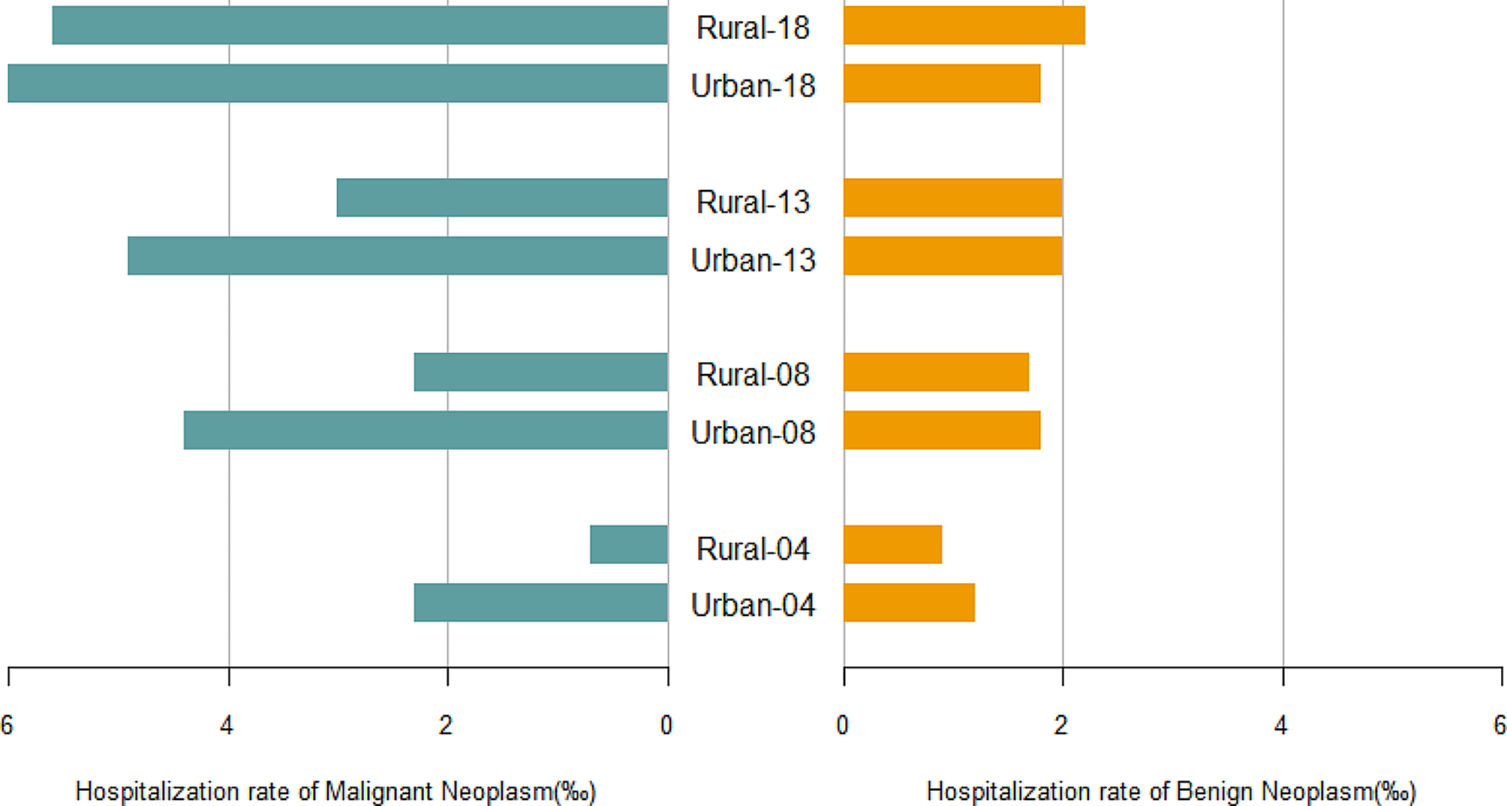
Bar chart of malignant neoplasm and benign neoplasm in China (2004, 2008, 2013, 2018)

**Fig. 3:**
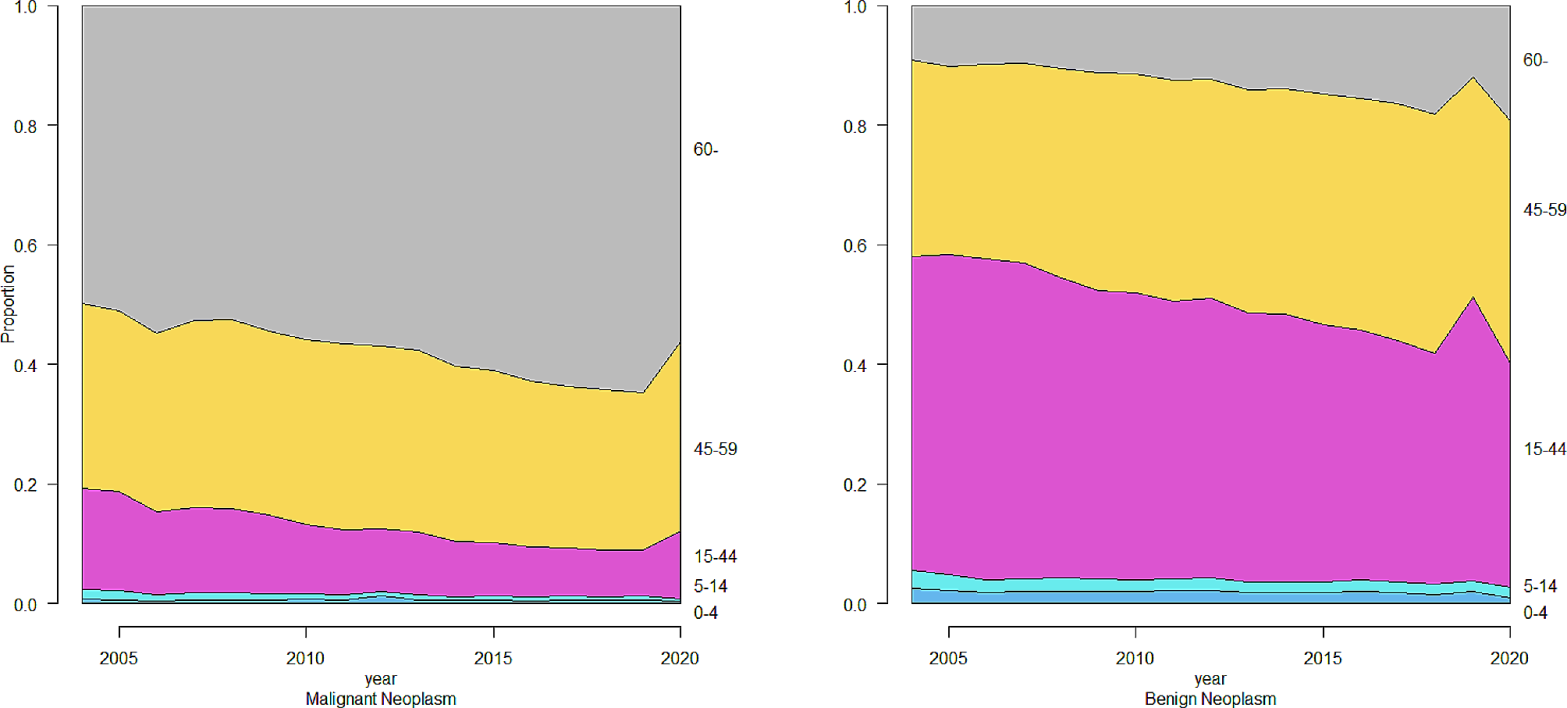
Trends in the distribution of age in hospitalization rate during 2004–2020. The patients were divided into five age groups: ≤4 years old, 5–14 years old, 15–44 years old, 45–59 years old, and ≥ 60 years old

We noted an increasing trend for the total number of hospitalizations from 245,645 in 2004 to 3,528,403 in 2020 (P for trend < 0.001) and benign neoplasm from 131,603 in 2004 to 1,785,741 in 2020 (P for trend < 0.001) (Fig. 1). The number of hospitalizations for malignant neoplasm and benign neoplasm in 2020 declined by 8.71% and 8.84%, respectively compared to 2019. The malignant neoplasm hospitalization rate increased from 1.1‰ to 5.8‰ and the benign neoplasm hospitalization rate increased from 1.0‰ to 2.0‰ during the study period (Fig. 2). Despite the increase in the number of hospitalizations and hospitalization rate, the in-hospital mortality rate due to malignant neoplasm and benign neoplasm decreased from 5.11% in 2004 to 2.87% in 2020 (P for trend < 0.001) and 0.14% in 2004 to 0.01% in 2020 (P for trend < 0.001), respectively (Fig. 1). In both urban and rural areas, the hospitalization rate of malignant neoplasms increased clearly and benign neoplasms increased slowly during the same period. It is worth noting that the gap between urban and rural hospitalization rates declined (Fig. 2). Trends in the distribution of age in hospitalization rate during 2004–2020 are shown in Fig. 3. The age distribution of malignant neoplasm and benign neoplasm did not change significantly in the 0–4 years old and 5–14 years old populations, while the proportion in ≥ 60 years old increased.

### Trends in malignant neoplasm and benign neoplasm-associated hospitalization cost and length of stay outcomes

**Fig. 4:**
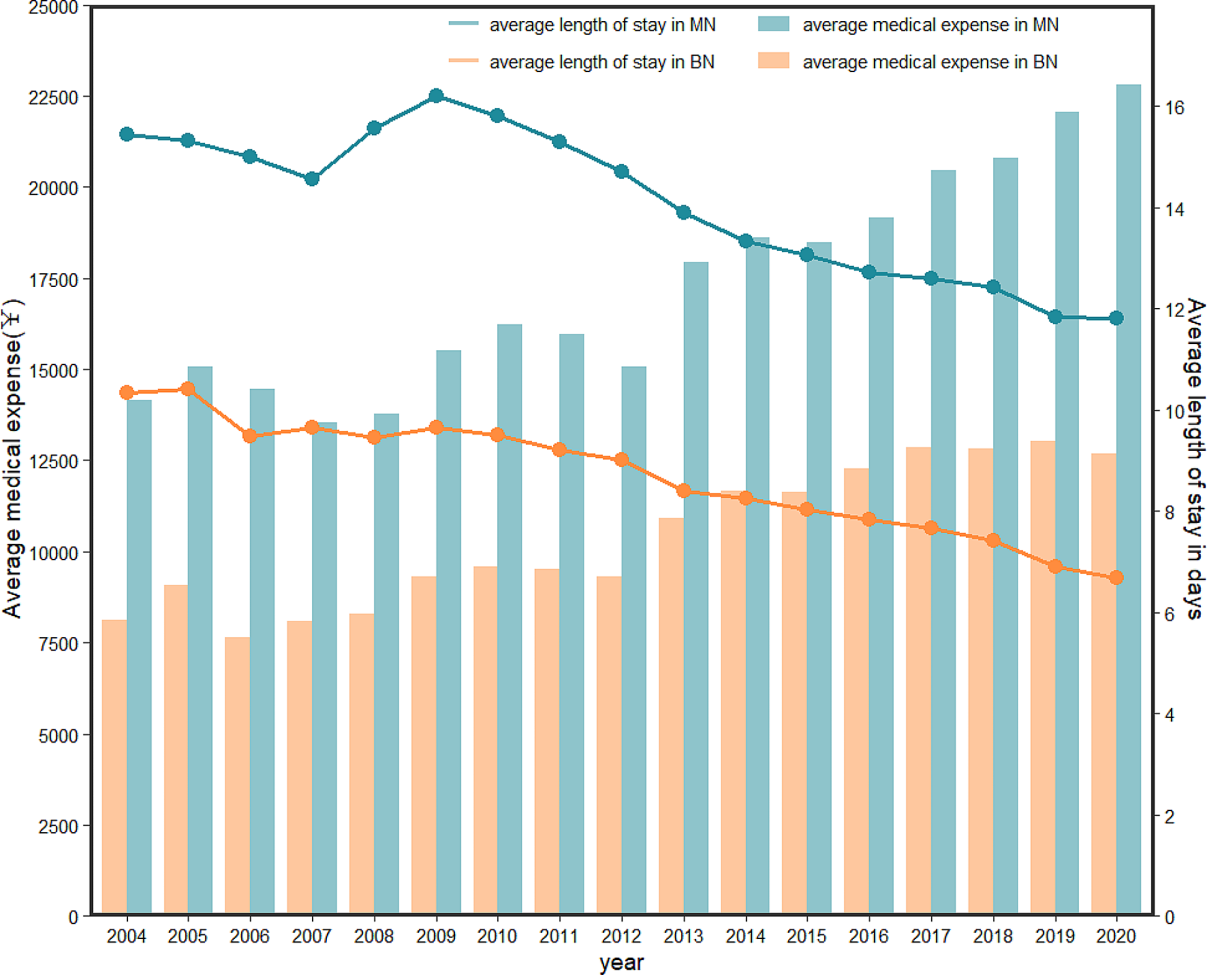
Trends of average medical expense and the average length of stay in malignant neoplasm and benign neoplasm-related. MN, malignant neoplasm; BN, benign neoplasm

Among all patients hospitalized with malignant neoplasm, the average cost per hospitalization significantly increased during the study period (from ￥14,136.59 in 2004 to ￥22,809.14 in 2020, P for trend < 0.001), adjusted for CPI (Fig. 4). However, the average LOS gradually decreased over the same period (from 15.44 days in 2004 to 11.82 days in 2020, P for trend < 0.001). In line with the trend of malignant neoplasm, the average cost per hospitalization increased significantly during the study period among all patients hospitalized with benign neoplasm (from ￥8,125.39 in 2004 to ￥12,695.82 in 2020, P for trend < 0.001), adjusted for CPI, this steady downward trend was observed in the average LOS (from 10.34 days in 2004 to 6.69 days in 2020, P for trend < 0.001) (Fig. 4).

## Discussion

China is the world’s largest developing country with an immense cancer burden [1, 14]. Compared with the United States and the United Kingdom, China has a lower incidence of cancer but a higher mortality and burden of disability-adjusted life years (DALYs) [5, 15]. To the best of our knowledge, this is the first recent study to examine the changing patterns of hospitalization status for neoplasms in China over the past 17 years, using a nationwide database.

This study noted the number of hospitalizations and hospitalization rates for neoplasms in China increased between 2004 and 2020. In 2020, 6.74% of all hospitalizations were for neoplasms [16]. However, the in-hospital mortality rate cause by malignant and benign neoplasms decreased from 5.11% to 0.14% in 2004 to 2.87% and 0.01% in 2020, respectively. This finding was consistent with that of another study that showed decreased in-hospital mortality and increased hospitalizations of pancreatic cancer patients in the US [17]. In addition, our study supported the results of two earlier Chinese studies. One demonstrated a decrease in age-standardized mortality rates of all cancers decreased from 2004 to 2018 [18]. The other displayed a decreased trend in most cancer deaths from 1990 to 2017, while cancer incidence increased sharply [19]. The results of studies in Spain, the United States and Australia differed from this finding. Hospitalization and hospital mortality rates for lung cancer in Spain showed a downward trend over the past 10 years [20]. Decreasing hospitalizations and mortality from gastric cancer with rising costs in the United States from 2003 to 2014 [21].

Contributing to the increase in hospitalization rates in China and the decrease in other countries are likely multifactorial. For example, the rising incidence of neoplasm, improvements in diagnostic and medical techniques, as well as increases in health insurance coverage and resident income for access to healthcare service [22]. In the last 30 years, the incidence of neoplasm has risen in China and decreased in the United States [22, 23]. From 1990 to 2019, the number of new cancer cases increased by 168.78% and the incidence rate increased by 22.21% in China [24].

Another possible explanation for this is the improvement in survival in China has been even greater, although still lower than in some developed countries. China’s 5-year relative survival for all cancers combined increased substantially from 30.9 to 40.5% between 2003 and 2015, while the United States improved from 63 to 68% between 1995 and 2018 [25, 23].

Besides, Roemer’s Law states an increase in the number of hospital beds per capita increases hospital utilization rates. Therefore, changes in hospitalization rates stem from the availability of hospital beds [26, 27]. In contrast, the incidence, hospitalization, mortality, and burden of cancer in Australia increased significantly between 1982 and 2014 [28]. Nevertheless, it has also been considered that increasing hospitalization rates could not worsen outcomes if patients who would benefit from hospitalization are instead discharged home [29].

Notably, hospitalizations for malignant and benign neoplasms decrease by 8.71% and 8.84%, respectively, in 2020 compared with 2019. This finding supports evidence from previous observations. The number of admissions and outpatient visits in China declined by 17.74% and 14.37%, respectively, in 2020 compared with the predicted values [30]. 54% fewer admissions through all neoplasms in Madrid in 2020 compared with the equivalent period in 2019 [31]. The COVID-19 pandemic had negative effects on health services access and utilization worldwide during the first wave of the pandemic [32, 33]. In this COVID-19 outbreak, the major risk for patients with cancer is the inability to receive necessary medical services because of the outbreak [34].

The estimated medical cost of hospitalization for malignant and benign neoplasms in pandocheum over 17 years was around￥706.2 billion [16]. The average cost per inpatient grew significantly during the study period, adjusted for CPI. During the same span, neoplasm patients’ average LOS did, however, gradually decline. Similarly, a US study found hospitalizations of pancreatic cancer increased over the last 10 years, whereas mean LOS and inpatient mortality decreased [35]. This could be a result of China’s more advanced diagnosis and treatment approaches, which have aided in the drop in in-hospital mortality and LOS. Despite improvements in health care and increasing funding for cancer control, the cancer burden in China remains at a high level.

Our study showed a gradual increase in hospitalization rates for neoplasms in both urban and rural areas. However, the gap between urban and rural areas has narrowed with assigning more health care to rural areas than before. Since China entered the market economy era in 1993, the socioeconomic gap between urban and rural areas has gradually narrowed, but differences in education, medical services, and insurance cancers status remain [6, 36, 37]. The hospitalization rates are still lower in rural areas than urban ones, although combined neoplasms incidence and mortality are significantly in rural areas than urban areas (213.6 vs. 191.5 cases and 149.0 vs. 109.5 deaths, respectively) [22]. This is also cause for concern.

As for age distribution, middle-aged and elderly groups accounted for most of neoplasm hospitalization. The proportion of neoplasm hospitalization in age group of 60 and above was an upward trend, telling that is aging and increasing burden on the elderly group.

In China, neoplasms continue to represent a heavy disease burden, both somatically and economically. The increasing cost should encourage healthcare policymakers and those involved in healthcare systems to develop more cost-effective approaches to neoplasm management, especially malignant neoplasm. For clinicians, the obvious decline in in-hospital mortality among neoplasm patients is encouraging and supports the view that neoplasm survival is preventable. There is still a need to raise awareness about healthy lifestyles through education and improve accessibility to curative and preventive healthcare services for all regardless of socioeconomic status or location.

### Limitations

This current study had several limitations: First, since the publicly available data did not report gender-specific hospitalization outcomes collection, and comparisons of trends in hospitalization between genders were not evaluated. Second, this study included data on pandocheum only, thus we have no information on those neoplasm patients admitted to other types of hospitals. In addition to pandocheum, hospitals belonging to the National Health Commission of the People’s Republic of China also include hospitals of Chinese medicine, hospitals of traditional Chinese and Western medicine, minority hospitals, specialized hospitals, and nursing homes. In 2020, the number of pandocheum had exceeded 20,000, and the number of inpatient admissions to pandocheum accounted for more than 75% of the total number of inpatient admissions to hospitals belonging to the National Health Commission across the country. However, this is still representative of the trend of hospitalization and outcomes across China. Third, this study did not report the reason for the patient’s hospitalization, such as receiving cancer-related treatment or treatment for other diseases or complications.

## Conclusions

Based on the analysis of nationally representative China data of hospitalized for neoplasm during 2004–2020, we found upward trends in the number of hospitalization, hospitalization rate, and medical cost in both malignant neoplasm and benign neoplasm. By contrast, we also observed substantial decreases in in-hospital mortality and LOS. The hospitalization rate gap between urban and rural areas is narrowed, although both malignant and benign neoplasms gradually increased in both urban and rural areas. The hospitalization ages of neoplasm were mainly concentrated in the middle-aged and elderly populations.

## Data Availability

The datasets used in this study can be accessed here: http://www.nhc.gov.cn/mohwsbwstjxxzx/tjzxtjcbw/tjsj_list.shtml.
